# Parathyroid Changes After RAI in Patients With Differentiated Thyroid Carcinoma

**DOI:** 10.3389/fendo.2021.671787

**Published:** 2021-05-27

**Authors:** Liu Xiao, Wenjie Zhang, Hongmei Zhu, Yueqi Wang, Bin Liu, Rui Huang, Lin Li

**Affiliations:** Department of Nuclear Medicine, West China Hospital of Sichuan University, Chengdu, China

**Keywords:** differentiated thyroid carcinoma, parathyroid hormone, serum calcium, serum phosphorus, radioactive iodine

## Abstract

**Objective:**

The purpose of this study was to investigate parathyroid hormone (PTH), serum calcium, phosphorus, and 25-hydroxyvitamin D (25-OH-VD) changes before and after radioactive iodine (RAI) in differentiated thyroid carcinoma (DTC) patients at different time points.

**Methods:**

A total of 259 DTC patients who received RAI were prospectively enrolled. We evaluated PTH, serum calcium, phosphorus, and 25-OH-VD levels at baseline pre-RAI, five days, six weeks, and six months post-RAI, respectively. We analyzed the risk factors of hypocalcemia at five days post-RAI.

**Results:**

The mean PTH, serum calcium and phosphorus values decreased five days post-RAI compared with pre-RAI (PTH 4.18 ± 1.23 pmol/L *vs*. 3.95 ± 1.41 pmol/L; calcium 2.27 ± 0.09 mmol/L *vs*. 2.20 ± 0.11 mmol/L; phosphorus 1.25 ± 0.17 *vs*. 0.98 ± 0.20 mmol/L, P < 0.05), and the differences were statistically significant. The mean 25-OH-VD levels did not significantly decrease at five days post-RAI. 21.2% (55/259) of patients had hypocalcemia at five days post-RAI, and all of them were given oral calcium supplements. At six weeks post-RAI, all of the above parameters were higher than those at five days post-RAI. Multivariate regression analysis showed that baseline pre-RAI serum calcium < 2.27 mmol/L, PTH < 4.18 pmol/L and negative ^99m^TcO_4_^-^ thyroid imaging were risk factors for hypocalcemia at five days post-RAI.

**Conclusion:**

For DTC patients with normal PTH and serum calcium levels at pre-RAI, their PTH, serum calcium, and phosphorus levels decreased at five days post-RAI. About one-fifth of patients could have hypocalcemia at five days post-RAI. Lower baseline pre-RAI serum calcium and PTH levels and negative ^99m^TcO_4_^-^ thyroid imaging were risk factors for hypocalcemia five days post-RAI.

## Introduction

The parathyroid gland is adjacent to the thyroid, located posterior to the thyroid. Theoretically, the β-rays released by radioactive iodine (RAI) for thyroid disease patients may affect the parathyroids. It has been reported that hyperparathyroidism occurs after RAI treatment of benign thyroid diseases (1–4). Szumowski et al. (5) observed parathyroid gland function changes after RAI treatment for benign thyroid disease. Their results indicated that PTH levels would be elevated for approximately ten months after RAI treatment. And the amplitude of increasing PTH is greater when the absorbed dose of thyroid tissue is higher.

The dose of RAI for differentiated thyroid carcinoma (DTC) therapy is significantly higher than that for benign thyroid diseases. Whether RAI affects DTC patients’ parathyroid gland function remains unclear. It has been reported that parathyroid hormone and serum calcium levels may decrease at six months post-RAI (6). However, some studies suggested that RAI did not affect parathyroid function (7, 8). Thus, the purpose of this study was to investigate PTH, serum calcium, phosphorus, and 25-OH-VD changes after RAI in patients with differentiated thyroid carcinoma (DTC) at different time points.

## Methods

### Patients Selection

We prospectively studied a total of 427 DTC patients who received RAI in the Department of Nuclear Medicine from West China Hospital of Sichuan University from December 2018 to December 2019. This study was approved by our institutional review board. Hypocalcemia and hypoparathyroidism were defined as serum calcium and serum PTH lower than the lower normal range limit in our institution (2.1 mmol/L and 1.6 pmol/L, respectively) (9). Inclusion criteria: patients with DTC who have undergone total thyroidectomy and have not consumed iodine-containing food and drugs within three weeks before RAI. Exclusion criteria: pregnancy and lactation, DTC complicated with other underlying severe diseases, a recent history of high iodine intake, parathyroid gland removal during surgery (according to the operation record), hypocalcemia, hypoparathyroidism, PTH level higher than upper normal range limit (PTH > 6.9 pmol/L) before RAI, recently taking calcium or vitamin D before RAI (within three weeks).

### RAI Therapy

RAI was initiated 3 to 6 months after total thyroidectomy. All the patients must discontinue levothyroxine replacement therapy for 3 - 4 weeks until the TSH level is > 30 mIU/L and must adhere to iodine restriction in their diet for at least two weeks before RAI. TNM staging was conducted according to the 8th edition of the American Joint Committee on Cancer (AJCC), and patients were stratified into low-risk, intermediate-risk, and high-risk groups according to the ATA guidelines released in 2015 (10). Postoperative ^99m^TcO_4_^-^ static thyroid imaging was performed one day before RAI was given. Thirty minutes after intravenous injection of 5 mCi ^99m^TcO_4_^-^, the imaging was acquired by a SPECT scanner. According to the comprehensive evaluation of the TNM stage, risk stratification, thyroid function, and imaging examination, RAI activity was established. Patients were given 100 mCi for remnant ablation and adjuvant therapy, and 150 - 250 mCi for treatment of known metastatic disease in our institution. Patients for remnant thyroid tissue ablation and adjuvant therapy, with suspected lymph node metastasis, lung metastasis, and bone metastasis, were given an RAI dose of 100mCi, 150 mCi, 200 mCi, and 250 mCi, respectively.

PTH, serum calcium, phosphorus, and 25-OH-VD were evaluated at baseline pre-RAI, five days, six weeks, and six months post-RAI, respectively. The PTH and 25-OH-VD values were measured using a fully automated electrochemiluminescent immunoassay analyzer (Cobas^®^ e601, Immunoassay Analyzer, Roche, Switzerland) with a measuring range of 0.127 - 530 pmol/L and 7.5 - 175 nmol/L, respectively. The normal reference range of PTH, 25-OH-VD, serum calcium, and phosphorus was 1.6 - 6.9 pmol/L, 47.7 - 144 nmol/L, 2.1 - 2.7 mmol/L and 0.81 - 1.45 mmol/L, respectively. Hypocalcemia symptoms (spasm or numbness in the hands, feet, and mouth) were inquired and recorded for each patient at five days post-RAI. Calcium supplementation status during hospitalization was also requested and recorded. Based on laboratory examination results at five days, patients with hypocalcemia were given oral calcium supplements, and patients with 25-OH-VD < 47.7 nmol/L were given calcitriol. According to the serum calcium levels, the serum calcium reduction was divided into three degrees: mild reduction (2 - 2.1 mmol/L), moderate reduction (1.9 - 2 mmol/L), and severe reduction (< 1.9 mmol/L).

### Follow-Up and Data Collection

After discharge, all the patients were informed to check PTH, serum calcium, phosphorus, and 25-OH-VD at six weeks and six months post-RAI, respectively. Data collection included the patients’ gender, age, TNM stage, risk stratification, number of RAI, number of surgeries, surgical methods (total thyroidectomy with or without central or lateral lymph node dissection), parathyroid transplantation, ^99m^TcO_4_^-^ thyroid imaging, RAI dose. Thyroid imaging results were considered positive or negative. Positive thyroid imaging showed ^99m^TcO_4_^-^ radioactive uptake in the thyroid bed area, and negative thyroid imaging indicated that no ^99m^TcO_4_^-^ uptake was found in the thyroid bed area.

We compared the changes of PTH, serum calcium, phosphorus, and 25-OH-VD levels between pre- and post-RAI. We also analyzed the risk factors for hypocalcemia at five days post-RAI.

### Statistical Analysis

Continuous variables were expressed as the mean ± standard deviation (SD) for variables with normal distributions or as the median and 25th to 75th percentiles (P25–75) for variables without normal distributions. Categorical variables are expressed as absolute numbers and percentages. Paired Student’s T-test was performed to analyze continuous data, including PTH, serum calcium, phosphorus, and 25-OH-VD at pre-RAI and post-RAI. Comparative analyses between normal serum calcium and hypocalcemia group were performed with an unpaired Student’s T-test for variables with normal distributions, the Mann-Whitney U-test for variables without normal distributions, and the Pearson Chi-Squared test for categorical variables. Multivariate linear regression analysis was used to evaluate risk factors for hypocalcemia at five days post-RAI. Statistical analysis was performed with SPSS software (SPSS, version 21.0; SPSS Inc., Chicago, IL, USA). A P-value < 0.05 was considered statistically significant.

## Results

**Figure 1:**
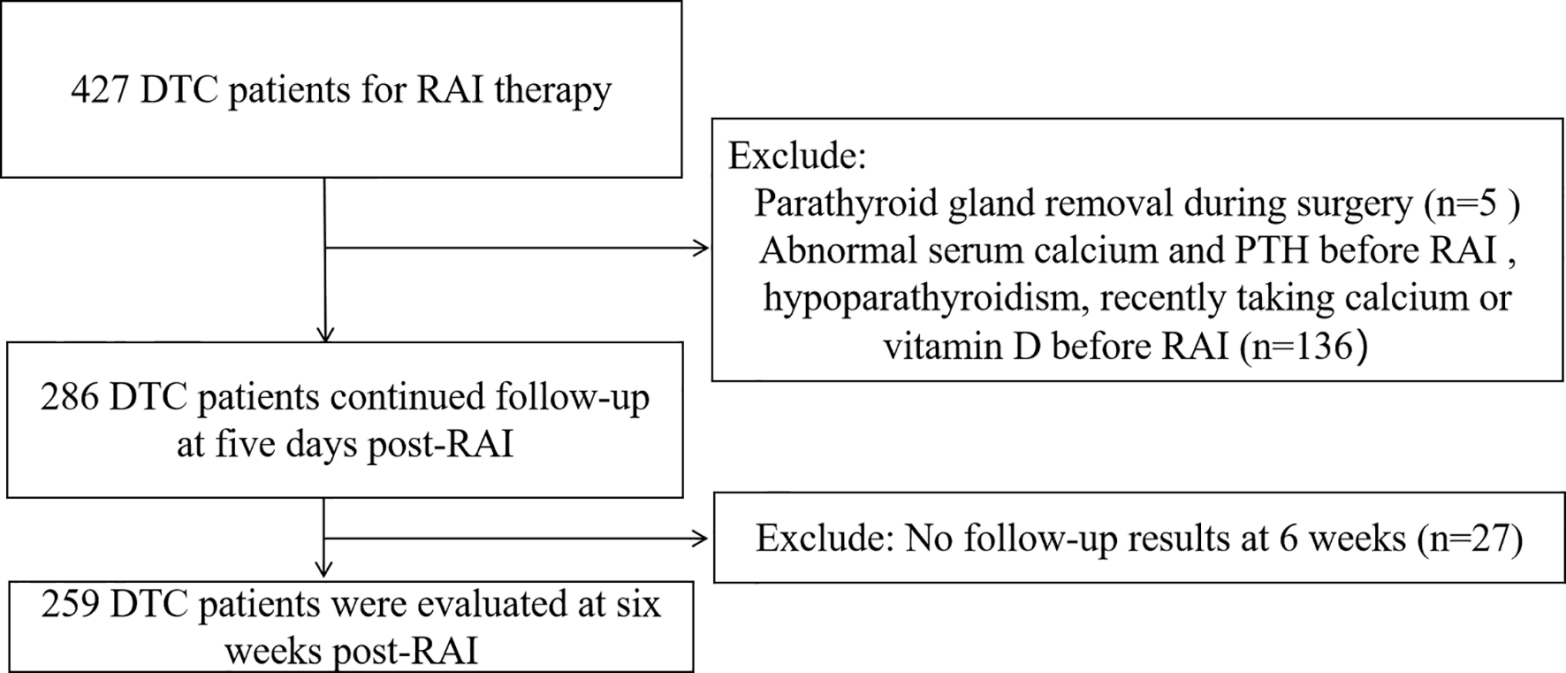
Flowchart of the study.

A total of 259 patients (94 males and 165 females) were eventually enrolled based on inclusion and exclusion criteria (**Figure 1**), with an average age of 40.6 ± 12.1 years. The study comprised 158 intermediate-risk patients and 101 high-risk patients. The mean PTH level at pre-RAI was 4.18 ± 1.23 pmol/L. The mean serum calcium level was 2.27 ± 0.09 mmol/L, ranging from 2.10 - 2.64 mmol/L. The mean serum phosphorus and mean 25-OH-VD levels were 1.25 ± 0.17 mmol/L and 51.90 ± 18.50 nmol/L, respectively. Other patients’ clinical characteristics information is shown in **Table 1**.

**Table 1:** Clinical characteristics of the patients.

Variables	N (%) or Value
**Age**	40.6 ± 12.1
**Gender** Male Female	94 165
**T stage** T1a + T1b T2 T3a + T3b T4a +T4b	136 (52.5%) 28 (10.8%) 44 (17.0%) 51 (19.7%)
**N stage** N0 N1a N1b	25 (9.7%) 124 (47.9%) 110 (42.4%)
**M stage** M0 M1	245 (94.6%) 14 (5.4%)
**AJCC stage** I II III IV	221 (85.3%) 25 (9.7%) 9 (3.5%) 4 (1.5%)
**Recurrence risk stratification** Intermediate risk High risk	158 (61%) 101 (39%)
**RAI dose** 100 mCi 150 mCi 200 mCi 250 mCi	166 (64.1%) 76 (29.3%) 16 (6.2%) 1 (0.4%)
**Number of RAI** 1 >1	234 (90.3%) 25 (9.7%)
**Number of surgeries** 1 >1	244 (94.2%) 15 (5.8%)
**Surgical method** TT + CLN TT + LLN	141 (54.4%) 118 (45.6%)
**Parathyroid transplantation** Yes No	163 (62.9%) 96 (37.1%)
**^99m^TcO_4_^-^ thyroid imaging** Positive Negative	53 (20.5%) 206 (79.5%)

TT, total thyroidectomy; CLN, central lymph node dissection; LLN, lateral lymph node dissection.

The mean PTH, serum calcium and phosphorus decreased at five days post-RAI compared with pre-RAI, and the differences were statistically significant (PTH 4.18 ± 1.23 pmol/L *vs*. 3.95 ± 1.41 pmol/L; serum calcium 2.27 ± 0.09 mmol/L *vs*. 2.20 ± 0.11 mmol/L; serum phosphorus 1.25 ± 0.17 *vs*. 0.98 ± 0.20 mmol/L, P < 0.05). In contrast to the mean PTH, mean serum calcium, and phosphorus levels, the mean levels of 25-OH-VD did not significantly decline at five days (51.90 ± 18.5 nmol/L *vs*. 49.71 ± 15.74 nmol/L, P = 0.147). Mean PTH, serum calcium, phosphorus, and 25-OH-VD levels at six weeks post-RAI showed an increasing trend compared with the values at five days post-RAI (PTH 4.62 ± 1.53 pmol/L *vs*. 3.95 ± 1.41 pmol/L; serum calcium 2.28 ± 0.11 mmol/L *vs*. 2.20 ± 0.11 mmol/L; 25-OH-VD 58.10 ± 19.01 nmol/L *vs*. 49.71 ± 15.74 nmol/L, P < 0.05), which basically recovered to the pre-RAI situation, but the mean serum phosphorus level was still lower than pre-RAI level (1.13 ± 0.18 mmol/L *vs*. 1.25 ± 0.17 mmol/L, P < 0.001) (**Table 2**).

**Table 2:** Comparison of PTH, serum calcium, phosphorus and 25-OH-VD values at pre-RAI, five days and six weeks post-RAI.

Group	Variable	Normal reference	Value	P (1, 2)	P (2, 3)	P (1–3)
1 2 3	PTH PTH (5 days) PTH (6 weeks)	1.6 - 6.9 pmol/L	4.18 ± 1.23 3.95 ± 1.41 4.62 ± 1.53	0.005	<0.001	<0.001
1 2 3	Calcium Calcium(5 days) Calcium(6 weeks)	2.1 - 2.7 mmol/L	2.27 ± 0.09 2.20 ± 0.11 2.28 ± 0.11	<0.001	<0.001	0.113
1 2 3	Phosphorus Phosphorus(5 days) Phosphorus(6 weeks)	0.81 - 1.45mmol/L	1.25 ± 0.17 0.98 ± 0.20 1.13 ± 0.18	<0.001	<0.001	<0.001
1 2 3	25-OH-VD 25-OH-VD(5 days) 25-OH-VD(6 weeks)	47.7 - 144 nmol/L	51.90 ± 18.5 49.71 ± 15.74 58.10 ± 19.01	0.147	<0.001	<0.001

**Figure 2:**
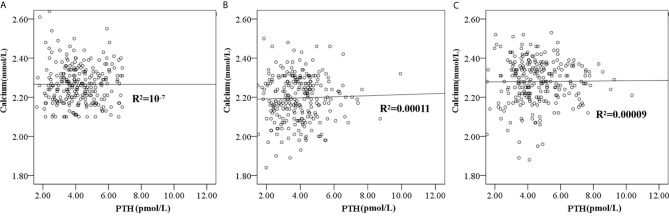
The scatter plot showed the individual simultaneous PTH and serum calcium at baseline pre-RAI **(A)**, five days post RAI **(B)** and six weeks **(C)** post-RAI.

Only three patients’ PTH values were less than 1.6 pmol/L (ranging from 1.35 to 1.51 pmol/L), and seven patients’ PTH values were higher than 6.9 pmol/L (ranging from 7.06 to 12.79 pmol/L) at five days post-RAI. PTH recovered to the normal reference range at six weeks post-RAI, except in 25 patients whose PTH was higher than 6.9 pmol/L (ranging from 6.96 - 10.35 pmol/L). Scatter plots showed individual simultaneous PTH and serum calcium at baseline pre-RAI, five days, and six weeks post-RAI (**Figure 2**). A total of 100 patients were re-checked PTH at the 6-month follow-up post-RAI. The mean PTH levels were 4.54 ± 2.28 pmol/L within the normal reference range.

Five days post-RAI, 21.2% (55/259) of the patients had hypocalcemia, including 78.2% (43/55), 18.2% (10/55), 3.6% (2/55) patients with mild, moderate, and severe reduction, respectively. During hospitalization for RAI, 9.6% (25/259) of patients showed hypocalcemia symptoms. Patients with hypocalcemia (n = 55) and 25-OH-VD level < 47.7 (n = 115) nmol/L five days post-RAI were given oral calcium and calcitriol supplements, respectively, until the next follow-up at six weeks post-RAI. At six weeks post-RAI, 6.2% (16/259) of patients had hypocalcemia, including 75% (12/16), 12.5% (2/16), 12.5% (2/16) with mild, moderate, and severe reduction, respectively. 42.9% (111/259) of patients re-checked their serum calcium at six months post-RAI. The mean serum calcium level (2.29 ± 0.11 mmol/L) recovered to the pre-RAI level six months post-RAI. Among them, four patients had hypocalcemia with mild reduction.

### Effect of RAI Dose on PTH, Serum Calcium, Phosphorus, and 25-OH-VD

According to the RAI dose, we divided DTC patients into two groups: the 100 mCi and > 100 mCi groups. The 100 mCi group had 166 patients. The > 100 mCi group included 76,16 and 1 for RAI of 150 mCi 200 mCi and 250 mCi, respectively. There were no significant differences in the mean PTH, serum phosphorus, or 25-OH-VD levels between the two groups at either pre- or post-RAI. At five days post-RAI, the mean serum calcium in the 100 mCi group was higher than that in the > 100 mCi group (2.21 ± 0.11 mmol/L *vs*. 2.17 ± 0.11 mmol/L, P = 0.004, respectively) (**Table 3**).

**Table 3:** Comparison of PTH, serum calcium, phosphorus and 25-OH-VD values at pre-RAI and post-RAI between the 100 mCi group and larger 100 mCi group.

Variable (reference range)	100 mCi group	> 100mCi group	P value
**Pre-RAI**			
PTH (1.6 - 6.9 pmol/L)	4.17 ± 1.24	4.21 ± 1.23	0.809
Calcium (2.1 - 2.7 mmol/L)	2.27 ± 0.10	2.26 ± 0.09	0.332
Phosphorus (0.81 - 1.45mmol/L)	1.25 ± 0.16	1.24 ± 0.18	0.308
25-OH-VD (47.7 - 144 nmol/L)	50.56 ± 17.65	54.31 ± 19.80	0.118
**5 days post-RAI**			
PTH (1.6 - 6.9 pmol/L)	3.89 ± 1.45	4.06 ± 1.34	0.338
Calcium (2.1 - 2.7 mmol/L)	2.21 ± 0.11	2.17 ± 0.11	0.004
Phosphorus (0.81 - 1.45mmol/L)	0.97 ± 0.20	0.98 ± 0.22	0.765
25-OH-VD (47.7 - 144 nmol/L)	48.78 ± 14.69	51.38 ± 17.43	0.202
**6 weeks post-RAI**			
PTH (1.6 - 6.9 pmol/L)	4.63 ± 1.61	4.62 ± 1.37	0.999
Calcium (2.1 - 2.7 mmol/L)	2.28 ± 0.11	2.28 ± 0.11	0.954
Phosphorus (0.81 - 1.45mmol/L)	1.41 ± 0.18	1.10 ± 0.19	0.112
25-OH-VD (47.7 - 144 nmol/L)	56.69 ± 18.53	60.56 ± 19.64	0.136

### Risk Factor Analysis of Hypocalcemia at Five Days Post-RAI

All the patients were divided into a normal serum calcium group and hypocalcemia group (< 2.1 mmol/L) according to serum calcium levels at five days post-RAI. The mean pre-RAI PTH and serum calcium levels in the normal serum calcium group were higher than those in the hypocalcemia group (4.30 ± 1.23 pmol/L *vs*. 3.74 ± 1.19 pmol/L, P = 0.03 and 2.28 ± 0.09 mmol/L *vs*. 2.19 ± 0.06 mmol/L, P < 0.001, respectively). The surgical method (total thyroidectomy + lateral lymph node dissection), RAI dose > 100 mCi, number of RAI > 1, and negative ^99m^TcO4^-^ thyroid imaging rate in hypocalcemia group were higher than in the normal serum calcium group (60% *vs*. 41.7%, P = 0.015; 47.3% *vs*. 32.8%, P = 0.048; 18.2% *vs*. 7.4%, P = 0.016; 90.9% *vs*. 76.5%, P = 0.018, respectively). There were no significant differences in mean serum phosphorus, TSH, or 25-OH-VD levels at pre-RAI, age, gender, number of surgeries, TNM staging, AJCC staging, risk stratification, or parathyroid transplantation between the hypocalcemia and normal serum calcium groups (**Table 4**).

**Table 4:** Comparison of baseline related parameters between hypocalcemia group and normal serum calcium group at five days post-RAI.

Variable (reference range)	Hypocalcemia group	Normal serum calcium group	P value
**PTH** (1.6 - 6.9 pmol/L)	3.74 ± 1.19	4.30 ± 1.23	0.03
**Calcium** (2.1 - 2.7 mmol/L)	2.19 ± 0.06	2.28 ± 0.09	< 0.001
**Phosphorus** (0.81 - 1.45 mmol/L)	1.28 ± 0.17	1.24 ± 0.17	0.109
**25-OH-VD** (47.7 - 144 nmol/L)	55.04 ± 20.79	51.06 ± 17.80	0.158
**TSH** (0.27 - 4.2 mIU/L)	77.22 (61.53-100)	80.24 (56.23-100)	0.457
**Age** >55 <55	5 (9.1%) 50 (90.9%)	29 (14.2%) 175 (85.8%)	0.318
**T stage** T1a + T1b T2 T3a + T3b T4a + T4b	22 (40%) 9 (16.4%) 10 (18.2%) 14 (25.4%)	114 (55.9%) 19 (9.3%) 34 (16.7%) 37 (18.1%)	0.151
**N stage** N0 N1a + N1b	3 (5.5%) 52 (94.5%)	22 (10.8%) 182 (89.2%)	0.235
**M stage** M0 M1	50 (90.9%) 5 (9.1%)	195 (95.6%) 9 (4.4%)	0.305
**AJCC stage** I II III IV	48 (87.3%) 5 (9.1%) 1 (1.8%) 1 (1.8%)	173 (84.8%) 20 (9.8%) 8 (3.9%) 3 (1.5%)	0.867
**Risk stratification** Intermediate High	29 (52.7%) 26 (47.3%)	129 (63.2%) 75 (36.8%)	0.156
**RAI dose** 100 mCi >100 mCi	29 (52.7%) 26 (47.3%)	137 (67.2%) 67 (32.8%)	0.048
**Number of RAI** 1 >1	45 (81.8%) 10 (18.2%)	189 (92.6%) 15 (7.4%)	0.016
**Number of surgeries** 1 >1	51 (92.7%) 4 (7.3%)	193 (94.6%) 11 (5.4%)	0.838
**Surgical method** TT + CLN TT + LLN	22 (40%) 33 (60%)	119 (58.3%) 85 (41.7%)	0.015
**Parathyroid transplantation** Yes No	32 (58.1%) 23 (41.9%)	131 (64.2%) 73 (35.8%)	0.676
**Thyroid imaging** Positive Negative	5 (9.1%) 50 (90.9%)	48 (23.5%) 156 (76.5%)	0.018

TT, total thyroidectomy; CLN, central lymph node dissection; LLN, lateral lymph node dissection.

Multivariate regression analysis showed that negative ^99m^TcO4^-^ thyroid imaging (95% CI: 1.095 - 8.754, P = 0.033), pre-RAI serum calcium < 2.27 mmol/L (95% CI: 3.092 - 14.466, P < 0.001), and pre-RAI PTH < 4.18 pmol/L (95% CI: 1.067 - 4.335, P = 0.032) were risk factors for hypocalcemia at five days post-RAI. T stage, risk stratification, RAI dose, number of RAI, and surgical method were not risk factors for hypocalcemia at five days post-RAI (**Table 5**).

**Table 5:** Multivariate regression analysis of the risk factors for hypocalcemia at five days post-RAI.

Variable	OR	95% CI	P value
T stage (T4a + T4b)	0.866	0.54 - 1.388	0.549
Risk stratification (high risk)	1.083	0.33 - 3.55	0.895
RAI dose > 100 mCi	1.122	0.485 - 2.596	0.788
Number of RAI > 1	0.481	0.156 - 1.485	0.203
Surgical method (TT + LLN)	0.497	0.246 - 1.003	0.051
Negative thyroid imaging	3.096	1.095 - 8.754	0.033
Pre-RAI calcium < 2.27 mmol/L	6.689	3.092 - 14.466	< 0.001
Pre-RAI PTH < 4.18 pmol/L	2.152	1.067 - 4.335	0.032

TT, total thyroidectomy; LLN, lateral lymph node dissection.

## Discussion

The effect of RAI on parathyroid remains unclear. In clinical practice, we observed that some DTC patients had hypocalcemia symptoms such as fatigue and numbness in their hands and feet during hospitalization for RAI. This may suggest that RAI have a “short-term” impact on parathyroid for these DTC patients. Thus, we performed a prospective study to investigate the effect of RAI on DTC patients’ PTH, serum calcium, and phosphorus at different time points.

Our study results indicated that mean PTH and serum calcium showed a slightly decreasing trend at five days post-RAI. These parameters recovered to the pre-RAI levels at six weeks post-RAI. The reason for the impairment of DTC patients’ parathyroid function after RAI is still unclear. It has been reported that a non-targeted effect of ionizing radiation may cause diminished parathyroid function after RAI, called a “bystander effect” on adjacent cells (6, 11). Guven et al. reported that PTH decreased in the short term but generally did not lead to hypocalcemia (6). However, some studies concluded that RAI did not affect the DTC patients’ parathyroid gland function (7, 8). This may partially because their shortest research time point was one month post-RAI. Since similar to their findings, PTH levels at about one month post-RAI could recover to the pre-RAI levels.

Szumowski et al. reported that RAI for hyperthyroidism caused transient hyperparathyroidism (5). They speculated that post-radiation inflammation of the parathyroid gland may cause an excessive release of PTH in the parathyroid cells. They also found that when the thyroid-absorbed dose was higher, the impact on parathyroid gland function was more significant (5). It has been reported that the RAI dose rates for hyperthyroid patients were higher than those for thyroid cancer patients (12). The high thyroid absorbed dose in hyperthyroid patients may cause transient hyperparathyroidism after RAI therapy.

About one-fifth of DTC patients had hypocalcemia (< 2.1 mmol/L) at five days post-RAI, and about one-tenth of them had symptoms. Multivariate regression analysis showed that negative ^99m^TcO_4_^-^ thyroid imaging was a risk factor for hypocalcemia at five days post-RAI. ^99m^TcO_4_^-^ thyroid imaging results can be used to predict the residual level of thyroid tissue after surgery (13, 14). Negative ^99m^TcO_4_^-^ thyroid imaging means less residual thyroid tissue after surgery, indicating that the surgical removal may be more thorough. In this case, it is more likely to cause accidental impairment of parathyroid reserve function and further causing hypocalcemia after RAI.

We found that the mean pre-RAI PTH and serum calcium levels in the hypocalcemia group at five days post-RAI were lower than those in the normal serum calcium group. This may indicate that the reserved parathyroid glands function in hypocalcemia patients after RAI was lower than that in patients with normal serum calcium (15). The results also showed that low pre-RAI serum calcium and PTH values were risk factors for hypocalcemia five days post-RAI by multivariate regression analysis. The mean serum calcium level recovered to the pre-RAI level at six weeks. All the hypocalcemia patients five days post-RAI were given oral calcium supplements. This may be one reason for the recovery of serum calcium at six weeks post-RAI. It is a pity that we cannot exclude spontaneous recovery of serum calcium due to the lack of control patients who did not take oral calcium supplements five days post-RAI.

The mean serum phosphorus decreased at five days post-RAI. At six weeks post-RAI, it was still lower than the pre-RAI situation. Theoretically, decreasing PTH would cause increasing phosphorus levels. The exact mechanism for decreased serum phosphorus at five days post-RAI is not clear. This may be explained by other mechanisms, such as fluid accumulation.

This study showed that some DTC patients have hypocalcemia five days post-RAI. The relevant risk factors of hypocalcemia at five days post-RAI may help clinicians watch out for these high-risk patients. Before RAI, we may even remind them they might become hypocalcemia after RAI and remember to take calcium supplements on time.

Our study had some weaknesses. First, the studied time range was six months. Future studies should extend the follow-up time range to determine whether RAI has a long-term impact on parathyroid function. Second, this study only analyzed common clinical factors and could not exclude decreasing serum calcium caused by other combined factors. Third, this study lacks controls for the patients who had hypocalcemia at five days post-RAI but did not take calcium. Last, 62.9% of patients in our study had undergone parathyroid gland transplantation. This may cause a selection bias. However, a recent study showed that parathyroid transplantation’s different ratios did not influence parathyroid function at post-RAI (16).

## Conclusion

For DTC patients with normal PTH and serum calcium levels at pre-RAI, their PTH, serum calcium, and phosphorus levels decreased at five days post-RAI. About one-fifth of patients could have hypocalcemia at five days post-RAI. Lower baseline pre-RAI serum calcium and PTH levels and negative ^99m^TcO_4_^-^ thyroid imaging were risk factors for hypocalcemia five days post-RAI.

## Data Availability Statement

The raw data supporting the conclusions of this article will be made available by the authors, without undue reservation.

## Ethics Statement 

The authors are accountable for all aspects of the work in ensuring that questions related to the accuracy or integrity of any part of the work are appropriately investigated and resolved. The study protocol was reviewed and approved by the institutional review board.

## Author Contributions 

LL and WZ conceived and designed this study. LX, HZ, and YW collected the data. LX drafted the paper and WZ edited it. BL, RH, and LL reviewed and edited the paper. All authors contributed to the article and approved the submitted version.

## Funding

The research was supported by “1.3.5 project for disciplines of excellence, West China Hospital, Sichuan University (ZYGD18016)”.

## Conflict of Interest

The authors declare that the research was conducted in the absence of any commercial or financial relationships that could be construed as a potential conflict of interest.
